# Genome‐wide DNA methylation profiling of CpG islands in a morpholino anthracycline derivative‐resistant leukemia cell line: p38*α* as a novel candidate for resistance

**DOI:** 10.1002/prp2.285

**Published:** 2016-12-29

**Authors:** Takeshi Asano, Hidehiko Narazaki, Atsushi Fujita

**Affiliations:** ^1^ Department of PediatricsNippon Medical SchoolTokyoJapan

**Keywords:** Drug resistance, key node search, leukemia, methylation array, morpholino anthracycline, p38*α*

## Abstract

Effective leukemia treatment is seriously hampered by drug resistance. We previously showed that aberrant methylation of the topoisomerase II *α* gene causes altered gene expression and acquired drug resistance in etoposide‐resistant leukemia cells. In this study, we analyzed the genome‐wide methylation status in resistant leukemia cells. We used MX2, which is a morpholino anthracycline derivative that functions as a topoisomerase II *α* inhibitor. We established a human myelogenous leukemia cell line (K562/P) and a related cell line with resistance to MX2 (K562/MX2). Using these cell lines, we investigated the genome‐wide methylation status, compared expression profiles with a microarray, and analyzed the data using Gene Ontology and key node analysis. We demonstrate that the MX2‐resistant cell line was globally hypermethylated. Gene Ontology analysis identified genes involved in the immunological response and gene silencing that were responsible for methylation‐related altered gene expression in drug‐resistant cells. Key node analysis showed that p38*α* mitogen‐activated protein kinase was a novel enzyme involved in MX2‐related resistance. p38 kinase activity in resistant cells was increased compared to MX2‐sensitive parent cells. Blocking p38*α* activity using inhibitors and p38*α* knock down with small interfering RNA restored the sensitivity to MX2 in resistant cells with a decrease in p38 kinase activity as well as decreased expression of p38*α* mRNA and phosphorylated p38*α* protein. These findings may lead to a new strategy for treatment of drug‐resistant leukemia cells.

**Abbreviations**
ADR: adriamycin
FDR: False discovery rate
GO: Gene Ontology
MAPK: mitogen‐activated protein kinase
MX2: 3′‐deamino‐3′‐morpholino‐13‐deoxo‐10‐hydroxycarminomycin hydrochloride
Topo: Topoisomerase

## Introduction

Effective leukemia therapy is hampered by drug resistance, which is a serious problem that results from various mechanisms. Pediatric leukemia patients with cancer cells that show resistance to antileukemic agents in vitro have a more dismal prognosis than patients with drug‐sensitive leukemia cells (Holleman et al. 2004). The pattern of gene expression in leukemia cells with acquired resistance to standard therapy is probably substantially different compared to leukemia cells prior to the initiation of treatment because drug‐resistant subpopulations grow selectively as treatment progresses. Nevertheless, how cells acquire drug resistance is unclear. Many signaling pathways and genes that may affect the response of leukemia cells to therapy have been identified. Because many factors are involved in the mechanism of acquisition of drug resistance, the “one gene: one outcome” hypothesis cannot adequately explain acquired resistance in leukemia (Glasspool et al. 2006). Thus, multiple mechanisms and multiple genes rather than a single pathway or gene likely mediate acquired resistance.

Aberrant methylation, including genome‐wide hypomethylation and regional hypermethylation of promoters for genes such as tumor suppressors, is one mechanism of tumorigenesis (Eden et al. 2003). Methylation of eukaryotic DNA occurs at CpG‐enriched promoters. Such epigenetic changes are critically involved in acquisition of drug resistance, which is mediated by changes in gene expression that occur following chemotherapy but that are not caused by genetic mutations. Wei et al. (2003) used drug‐resistant cell lines and differential methylation hybridization and showed many differences in CpG island methylation and epigenetic regulation after drug treatment. However, this group did not determine which genes were aberrantly methylated in resistant cells. In general, recent studies including ours have investigated only a few CpG island markers in methylation‐related drug resistance (Asano et al. 2005).

3′‐deamino‐3′‐morpholino‐13‐deoxo‐10‐hydroxycarminomycin hydrochloride, also known as KRN 8602 (MX2), is a new morpholino anthracycline derivative that acts as a topoisomerase (Topo) II*α* inhibitor and is cytotoxic to tumor cells (Watanabe et al. 1988). MX2 is highly lipophilic and easily passes through the cell membrane in a P‐glycoprotein‐independent manner (Watanabe et al. 1988). The antitumor effects of MX2 are superior to those of adriamycin (ADR). MX2 is toxic to mouse and human tumor cell lines as well as multidrug‐resistant tumor cell lines that express high levels of P‐glycoprotein (Watanabe et al. 1991). MX2 may thus be useful for eradicating multidrug‐resistant tumors. By continuously exposing cells grown in suspension to increasing amounts of MX2, we previously established the MX2‐resistant human myelogenous leukemia cell line K562/MX2, which is derived from the parent cell line K562/P (Asano et al. 2005). K562/MX2 cells show lower levels of Topo II*α* mRNA and protein, and the Topo II*α* gene in these cells is aberrantly methylated at CpG islands. Thus, drug resistance in K562/MX2 cells may be due to aberrant methylation (Asano et al. 2005). We therefore next investigated the relationship between global gene expression and methylation in drug‐resistant cells and identified genes that confer resistance. High‐throughput methylation analysis of multiple CpG sites can be performed with the GoldenGate Methylation BeadArray (Illumina Inc. Tokyo, Japan) (Ang et al. 2010). Here, we evaluated the genome‐wide methylation status using the methyl array, compared gene expression profiles using microarray, and analyzed the entire profile of altered gene expression with methylation using Gene Ontology (GO) analysis. We found that resistant cells were hypermethylated in whole genes, and that genes involved in gene silencing and the immunological response were most critical for methylation‐related altered gene expression. In addition, using key node analysis, p38*α* mitogen‐activated protein kinase (MAPK) was identified as a novel enzyme that may mediate MX2‐related resistance. In addition to the K562 cell line, we also established a lymphoblastic leukemia cell line with resistance to MX2 (BALL/MX2). Compared to sensitive cells, p38 kinase activity in both resistant cell lines was increased. Blocking p38 kinase activity and phosphorylated p38*α* protein with SB203580 or SB20190, which are specific inhibitors of p38 MAPK, or using siRNA to knock down p38*α* mRNA expression, restored the sensitivity to MX2 in resistant cells, concomitant with decreased expression of p38*α* mRNA, phosphorylated protein, and kinase activity.

## Materials and Methods

### Reagents

We used the hydrochloride form of MX2 (Watanabe et al. 1988, 1991). ADR, etoposide, vincristine, and dimethyl sulfoxide, were purchased from Wako Pure Chemical Industries, Ltd. (Osaka, Japan). Phosphate‐buffered saline without metal salt solution (PBS (−)) was purchased from Nissui (Tokyo, Japan). RPMI 1640, Hanks' balanced salt solution without Ca^2+^ or Mg^2+^ (HBSS), fetal calf serum, and gentamicin were purchased from Life Technologies, Inc. (Gaithersburg, MD). 5‐Aza‐2′‐deoxycytidine was purchased from Sigma Aldrich Japan (Tokyo, Japan). SB203580 (4‐(4‐fluorophenyl)‐2‐(4‐methylsulfinylphenyl)‐5‐(4‐pyridyl)1H‐imidazole) and SB202190 (4‐(4‐fluorophenyl)‐2‐(4‐hydroxyphenyl)‐5‐(4‐pyridyl)1H‐imidazole), which are p38 MAPK inhibitors, and SB202474 (4‐Ethyl‐2(p‐methoxyphenyl)‐5‐(4′‐pyridyl)‐IH‐imidazole), which is a negative control, were purchased from Calbiochem (Tokyo, Japan). siRNAs were obtained from Ambion (Carlsbad, CA).

### Cell lines

Parental cell lines (K562/P, human myelogenous leukemia and BALL‐1, human B‐cell lymphoblastic leukemia) were purchased from RIKEN (Tsukuba, Japan). BALL‐1 (BALL) cell line is established from typical human B‐cell leukemia (male) (Miyoshi et al. 1977). K562 cell line is established from pleural effusion with chronic myelogenous leukemia of 53 years old female, which is sensitive to NK cell and can differentiate to erythroid cells (Lozzio and Lozzio 1975). The MX2‐resistant cell line was established with limiting dilution using continuous exposure to increasing amounts of MX2 (Asano et al. 2005). MX2‐resistant cells were cultured in the absence of MX2 for 2 weeks before use in experiments. The MycoAlert^TM^ mycoplasma detection kit (Lonza Walkersville Inc., Tokyo, Japan) was used to confirm the absence of *Mycoplasma* organisms in all cell lines.

### Cytotoxicity assay

The MTT assay (CellTiter96 AQueaus One solution Cell Proliferation Assay, Promega Corp., Madison, WI) or trypan blue exclusion was used to determine cytotoxicity (Asano et al. 2005). Briefly, 1 × 10^5^ cells/mL were incubated with various concentrations of MX2, etoposide, ADR, or vincristine for 72 h. Viable cells were counted after performing the MTT assay or trypan blue staining. The combination index method (Zhao et al. 2004) was used to determine the synergistic effect of inhibitors and siRNAs plus MX2.

### 5‐Aza‐2′‐deoxycytidine treatment

Briefly, 1 × 10^5^ cells/mL (total 1 × 10^7^ cells) were grown for 72 h in a medium that included 10 *μ*mol/L 5‐Aza‐2′‐deoxycytidine. Fresh medium and drug were replaced daily.

### Isolation of genomic DNA and quality assessment

DNA was extracted from freshly harvested cells with the QIAamp DNA Mini Kit (Qiagen, Tokyo, Japan). Low‐percentage (0.5%) agarose gel electrophoresis and low‐power voltage were used to assess the quality of the extracted DNA. Genomic DNA was of sufficient quality when a high molecular weight band (<40 kb) was visible and when no strong low molecular weight band (<2.0 kb) was visible on the gel after 3 hours of electrophoresis. Alternatively, an OD260/280 between 1.8 and 2.0 indicated DNA of sufficient quality.

### Bisulphite conversion

The EZ DNA methylation kit (Zymo Research, Irvine, CA) was used for bisulphite conversion of genomic DNA with modifications for the Illumina Infinium Methylation Assay (Illumina, Tokyo, Japan). Briefly, 1 *μ*g genomic DNA was incubated with 5 *μ*L M‐Dilution buffer at 37°C for 15 min. Then, 100 *μ*L CT conversion reagent was prepared as described by the manufacturer and added to the mixture, which was incubated in a thermal cycler for 16 cycles at 95°C for 30 sec and 50°C for 60 sec. Aliquots of bisulphite‐converted DNA were added to 96‐column plates provided in the kit, and then desulphonated and purified as described by the manufacturer. These samples were used immediately for chip analysis, as described below.

### Illumina infinium human methylation27 beadchip

The Illumina Infinium Human Methylation27 BeadChip Kit was used for assays with bisulphite‐converted genomic DNA. This beadchip includes 27,578 CpG loci in >14,000 human RefSeq genes at single‐nucleotide resolution. The reagents included in the kit were used for chip processing and analysis of data. Briefly, 4 *μ*L bisulphite‐converted genomic DNA was denatured in 0.014 N sodium hydroxide and neutralized. DNA was then amplified using components included in the kit for 20–24 h at 37°C. After fragmentation, 12 *μ*L of each sample was loaded onto a 12‐sample chip, which was inserted into a hybridization chamber as described by the manufacturer. Samples were incubated at 48°C for 16–20 h, chips were washed with wash buffers from the kit, and then chips were incubated in a fluid flow‐through station for primer extension and stained using components provided in the kit. The iScan scanner (Illumina) was used for image processing of polymer‐coated chips. We used a cutoff level for detection of a *P* < 0.001, which is the most stringent criterion in the Illumina GoldenGate Methylation array. BeadStudio v3.0 software (Illumina) was used for data extraction. Methylation values for each CpG locus were expressed as a *β*‐value, which is a continuous value from 0 (completely unmethylated) to 1 (completely methylated) and is based on the following equation: *β*‐value = (signal intensity of methylation‐detection probe)/(signal intensity of methylation‐detection probe + signal intensity of non methylation detection probe). In accordance with the manufacturer's recommendation, results >0.15 as determined by the difference in the *β*‐value between two sets of groups were considered significant.

### RNA isolation, real‐time PCR, and microarray analysis

The Qiagen RNA Mini kit (Qiagen) was used for isolation of total RNA from each sample, and RNA integrity was confirmed following 1% agarose gel electrophoresis. Real‐time PCR analysis was performed as described (Yamanishi et al. 2015) using the following primers: p38*α* sense: 5′‐TGCCCGAGCGTTACCAGACC‐3′ and antisense: 5′‐CTGTAAGCTTCTGACATTTC‐3′. The Agilent Low RNA Input Fluorescent Linear Amplification kit (Agilent Technologies, Tokyo, Japan) was used for in vitro transcription in the presence of Cy3‐ and Cy5‐CTP. Next, 825 ng‐labeled complementary RNA from each pair was purified separately, combined, mixed with hybridization buffer prepared using the In Situ Hybridization Plus kit (Agilent Technologies), and added to the microarray. Samples were hybridized to an Agilent 4 × 44k Whole Human Genome microarray (G4112F; Agilent Technologies), which contains 43,376 coding and noncoding sequences from the human genome, in an Agilent G2545A hybridization oven. Hybridization and washing conditions were as described by the manufacturer in the protocol for oligonucleotide microarray hybridization (Agilent Technologies). Feature Extraction software (version 9.3; Agilent Technologies) and Spotfire software (version 8.0; Spotfire, Cambridge, MA) were used to analyze data from the Agilent G52565BA microarray scanner. Fluorescent spots on the microarrays were considered present or absent. Automatic recognition software (Feature Extraction version 9.3; Agilent Technologies) selected transcripts that were considered present, and quantile normalization was used to normalize data. Spots that failed quality control procedures (those with signal intensity <1.0) were excluded from additional analysis. The possibility of dye‐related bias in the microarray results was excluded with an algorithm included in the software that applied normalization factors (linear and lowness normalization). Data were imported into Excel files (Microsoft, Redmond, WA) for subsequent data and statistical analysis (Agilent Technologies provides details of these procedures).

### Integration of methyl array data and expression array data

After quantile normalization, we integrated the data from the methyl array and expression array to identify genes that were strongly related to methylation‐specific altered expression. We considered increased methylation as a difference in the *β*‐value of >0.15, decreased methylation as a difference in the *β*‐value of <0.15, increased expression as >1.5‐fold of the expression level, and decreased expression as <1‐ to 1.5‐fold of the expression level.

### GO analysis

Functional class scoring analysis based on Biobase Knowledge Library manual curation was used to determine GO classes that were differentially methylated in drug‐sensitive compared to drug‐resistant cells. A *P* value for comparison of drug‐sensitive and ‐resistant cells was computed for each gene in a GO class. Multiple testing corrections with the Benjamini and Hochberg false discovery rate (FDR) (Reiner‐Benaim 2007) were used to determine the set of *P* values for a GO class.

### Key node search algorithm

The search for signaling molecules (key nodes) in the network vicinity of a gene list can be performed based on only one gene list, or based on a primary gene list with an additional gene list as a secondary set. Genes in the secondary set were incorporated such that the key node algorithm goes through the elements of this gene set. The network path was attracted by the secondary genes, resulting in longer paths that are often cheaper than shorter paths if they include molecules from the secondary set. The algorithm is a feed‐forward‐based approach that transforms the original weights of the network into new weights. The weights of the resulting network reflect the desired attraction power.

Score: The significance score, used for ranking the key node results, counted the hits that were relative to the respective logarithmized volume *Vi* that was required to reach every hit.

Equation 1. Score calculation

$$S=N_{0}+\sum_{i=1}^{r}\frac{N_{i}-N_{i-1}}{1+\log(V_{i})}$$

Volume *V*_*i*_ = number of total compounds reachable from the key node within a distance.

Hits *N*_*i*_ = number of targets reached by key node k within distance *i*.

With increasing distance *i*, the volume *V*_*i*_ also increases. The maximum distance is limited by *r*.

#### FDR

Each individual key node was assigned an FDR value, which represents the probability that the observed rank or higher ranks was occupied by random chance and was estimated on‐the‐fly by random sampling. The ranking of the key nodes was defined by sorting them according to the score described above in descending order. All key nodes with an observed rank <200 were assigned an FDR value of 1.0 by definition, because their score was considered insufficient. Molecules with no hits were assigned the last rank because the score was 0 in this case.

#### *Z*‐score

In addition to the FDR, each key node was assigned a *Z*‐score, which measures the deviation of the observed rank of the key node from the expected rank in a random case. The *Z*‐score was divided by the standard deviation.

Equation 2. Z‐score calculation

$$Z=\frac{X-\mu }{\sigma \mu \varepsilon^{-}}$$

In this formula, the rank distribution was assumed to comply with normal distribution. Key nodes with a *Z*‐score >1.0 were considered significant.

Promoter analysis was performed using the online tool ExPlain 3.1 (http://explain.biobase-international.com/) for detection of overrepresented transcription factor‐binding sites (Stegmaier et al. 2011; Zawacka‐Pankau et al. 2011; Takahashi et al. 2015). For the analysis, we selected regions from 1000‐bp upstream to 100‐bp downstream of the transcription start site of each gene of absolute fold change >4 (Yes set) and <1.12 (No set). The vertebrate_h0.01 set of transcription factors matrix from the TRANSFAC database was used for scanning potential binding sites. We used all promoters of genes with *P* < 0.01. The high‐specific matrices with a Yes/No score >2.1, *P* < 0.05, and matched promoters *P* < 0.05 were selected with cutoffs from minSUM. The upstream analysis was performed with distance threshold value of 6 and FDR < 0.05 including expression/transregulation reaction and following curated chains.

### Western blot analysis

Cells were lysed in RIPA buffer (WAKO Pure Chemical Industries Ltd.) with proteinase (Sigma Aldrich) and phosphatase inhibitors or in 1× Laemmli buffer, and lysates were separated on a SDS‐PAGE gel. After transfer to membranes, blots were incubated with primary antibodies against phosphorylated p38*α* (Cell Signaling Technology, Inc., Danvers, MA) and GAPDH (Wako).

### p38 kinase assay

The CycLex p38 Kinase Assay kit (CycLex Co., Ltd., Nagano, Japan) was used for the p38 kinase assay.

### Transfection of siRNA

siRNAs that were used to knock down p38*α* expression and the corresponding negative control siRNA were purchased from Ambion (Tokyo, Japan). Cells (3 × 10^3^ cells/well) were plated in 96‐well plates and incubated for 24 h. Cells were then transfected with 5 pmol control or p38*α* siRNA using Lipofectamine Plus transfection reagent (Life Technologies, Tokyo, Japan). Twenty‐four hours post transfection, the treated cells were incubated with various concentrations of MX2 for 72 h and measured cytotoxicity. For confirming to knock down p38*α* expression, mRNA were extracted 24 h after transfection, and protein were extracted 96 h after transfection. To establish 100% survival, cells were incubated with vehicle containing Hiperfect alone. Assays were performed in triplicate, and at least three independent experiments were conducted for each condition.

### Methylation‐specific polymerase chain reaction analysis for p38*α*

For MSP analysis, genomic DNA was obtained and 300 ng of DNA per sample was treated as described before (Yamanishi et al. 2015). Primer pairs for MSP of p38*α* (*Homo sapiens* mitogen‐activated protein kinase 14 (MAPK14) gene, GenBank: EU332860.1) were designed based on methylated and unmethylated DNA sequences in the promoter region, as follows: p38*α*1M; pos. 1951‐2059: 5′‐TATATTGGGTAAAATTTCGGTTTTC‐3′, 5′‐AATACTCCCGTTCCAACTACTACG‐3′, and p38*α*1U; 5′‐ TATATTGGGTAAAATTTTGGTTTTTG‐3′, 5′‐ATACTCCCATTCCAACTACTACACC‐3′. p38*α*2M; pos. 4618‐4738: 5′‐GTCGGGTGTAGTGGTTTACGT‐3′, 5′‐TTTAATAAAAACGAAATTTCACCG‐3′, and p38*α*2U; 5′‐GGTTGGGTGTAGTGGTTTATGT‐3′, 5′‐TTAATAAAAACAAAATTTCACCATA‐3′. p38*α*3M; pos. 10992‐11210: 5′‐TTTAGTTTGGAGTGTAGTGGTACGA‐3′, 5′‐ AAAAACCGAAACAAAAAAATCG‐3′, and p38*α*3U; 5′‐ TTTAGTTTGGAGTGTAGTGGTATGA‐3′, 5′‐AAAAAACCAAAACAAAAAAATCACT‐3′. p38a4M; pos. 13929‐14207: 5′‐GTTTAGGTTGGGTGTAGTGGTTTAC‐3′, 5′‐TAAAACTAAATCTTACTCTATCGCC‐3′, and p38*α*4U; 5′‐TTAGGTTGGGTGTAGTGGTTTATGT‐3′, 5′‐TTTTAAAACTAAATCTTACTCTATCACC‐3′. Polymerase chain reaction was performed using the EpiScope MSP kit (Takara Biotechnology Company, Ohtsu, Japan) and the ABI Prism 7500 sequence detection system (Applied Biosystems). Amplification was performed with an initial denaturation at 95°C for 30 sec, 45 cycles of denaturation at 98°C for 5 sec, annealing at 55°C for 30 sec, and extension at 72°C for 60 sec. Subsequently, melting curve analysis was performed on PCR products.

### Apoptosis assay

Caspase 3/7 activity assays were determined using Caspase‐Glo 3/7 assay according to the manufacturer's instructions in 96‐well plates (Promega, Madison, WI). We used 10 *μ*mol/L SB202190, 10 *μ*mol/L SB203580, and 10 *μ*mol/L SB202474 as a pretreatment for 30 min. Then, 2.5 nmol/L of MX2 were added, and the cells were cultured for 1 h and performed caspase 3/7 activity assays.

### Statistical analysis

The Kruskal–Wallis *H* test was used for statistical analysis. When a significant difference was detected, we used the Mann–Whitney *U* test to calculate the significance of differences between each group. Bonferroni–Dunn correction was performed with uncorrected *P* values by multiplying them by the number of comparisons.

## Results

### Establishment and features of the MX2‐resistant cell line

MX2‐resistant cells were established with constant treatment of MX2‐sensitive cells with increasing concentrations of MX2. This subculture of MX2‐resistant cells is grown in the continuous presence of 100 nmol/L or 200 nmol/L MX2, but the phenotype remains stable after growth in medium without MX2 for more than 6 months. MX2‐resistant K562 cells (K562/MX2 cells) were resistant to both MX2 and ADR and also exhibited cross‐resistance to etoposide (Table 1).

**Table 1:** IC50 values for MX2, etoposide, adriamycin, and vincristine with or without 5‐Aza‐2′‐deoxycytidine (5AZ) treatment

	MX2 (nM)	Etoposide (nM)	Adriamycin (nM)	Vincristine (nM)	Carboplatin (*μ*M)
K562/P	30 ± 4	10 ± 4	20 ± 3	2.0 ± 2.1	20 ± 5.1
K562/P with 10 *μ*M 5AZ	29 ± 6	7 ± 4	15.0 ± 11.0	1.8 ± 2.9	22 ± 4.8
K562/MX2	200 ± 23^a^	94 ± 15^a^	150 ± 20^a^	2.3 ± 1.8	18 ± 6.0
K562/MX2 with 10 *μ*M 5AZ	46 ± 9^b^	8 ± 5^b^	130 ± 10.7	2.0 ± 1.0	20.5 ± 4.8

The IC50 values were calculated from the cytotoxicity of various drugs.

Data are reported as the mean ± standard deviation from five independent experiments.

a *P* < 0.05, Cytotoxicity in K562/P versus K562/MX2 cells.

b *P* < 0.05, Cytotoxicity in K562 cells with versus without 5‐Aza‐2′‐deoxycytidine treatment.

### DNA methylation patterns in drug‐sensitive and ‐resistant leukemia cells

The DNA methylation pattern was clearly different in drug‐sensitive and ‐resistant leukemia cells. After filtering, 19,663 genes were eligible for statistical analysis. Significant results were determined by a difference in the *β* > 0.15 between the two sets of groups. This analysis identified 4184 genes as differentially expressed between MX2‐sensitive and ‐resistant leukemic cells. Of these, 3229 were hypermethylated, and 955 were hypomethylated in drug‐resistant leukemia cells compared with the same genes in the parent cells (Tables 2, 3 Table S1). Lists of all expression profiles including full gene names and gene accession numbers are shown in Table S1. 5‐Aza‐2′‐deoxycytidine treatment produced slight changes in methylation values on hypermethylated genes in K562/MX2 cells (Table 2), but no effect on hypermethylated genes in K562/P cells (Table 3).

**Table 2:** Methylation profile in K562/MX2 cells and K562/P cells. List of 30 highly methylated genes in K562/MX2 cells compared to K562/P cells

Gene symbol	Difference KMX(−)‐P(−)	KMX(−). Beta	P(−). Beta	KMX(+). Beta	P(+). Beta	Description	GenBank accession
*LOC63928*	0.9727	0.9848	0.0121	0.9471	0.0096	Hepatocellular carcinoma antigen gene 520	NM_022097.1
*FLJ36046*	0.9391	0.9696	0.0305	0.8823	0.0207	Hypothetical protein LOC164592	NM_152612.2
*TSC22D3*	0.9389	0.9667	0.0278	0.9247	0.0253	TSC22 domain family; member 3 isoform 2	NM_004089.3
*TMEM22*	0.9357	0.9797	0.0439	0.9712	0.0209	Transmembrane protein 22	NM_025246.1
*HMHA1*	0.9338	0.9662	0.0324	0.9516	0.0340	Minor histocompatibility antigen HA‐1	NM_012292.2
*PLXND1*	0.9304	0.9487	0.0183	0.8712	0.0108	Plexin D1	NM_015103.1
*SLC25A22*	0.9266	0.9539	0.0274	0.8998	0.0199	Mitochondrial glutamate carrier 1	NM_024698.4
*FHIT*	0.9205	0.9446	0.0241	0.9417	0.0185	Fragile histidine triad gene	NM_002012.1
*RGL3*	0.9184	0.9627	0.0443	0.9448	0.0419	Ral guanine nucleotide dissociation stimulator‐like 3 isoform 2	XM_934610.1
*RASL10B*	0.9154	0.9406	0.0252	0.8588	0.0170	RAS‐like; family 10; member B	NM_033315.2
*RHCG*	0.9143	0.9273	0.0130	0.8476	0.0201	Rhesus blood group; C glycoprotein	NM_016321.1
*CRIP3*	0.9143	0.9587	0.0444	0.9346	0.0370	Cysteine‐rich protein 3	NM_206922.1
*NPB*	0.9095	0.9386	0.0291	0.8942	0.0273	Preproneuropeptide B	NM_148896.2
*H2AFY2*	0.9072	0.9504	0.0432	0.9387	0.2239	Core histone macroH2A2.2	NM_018649.1
*GRIN2D*	0.9062	0.9437	0.0375	0.8909	0.0487	*N*‐methyl‐d‐aspartate receptor subunit 2D precursor	NM_000836.1
*ARHGAP4*	0.8988	0.9757	0.0770	0.9623	0.0383	Rho GTPase‐activating protein 4	NM_001666.2
*DRD1IP*	0.8950	0.9254	0.0304	0.8854	0.0422	Dopamine receptor D1 interacting protein	NM_015722.2
*SYK*	0.8930	0.9656	0.0727	0.9392	0.0451	Spleen tyrosine kinase	NT_008470.18
*KIF6*	0.8921	0.9197	0.0276	0.8709	0.0258	Kinesin family member 6	NM_145027.3
*MC1R*	0.8914	0.9758	0.0844	0.9632	0.0365	Melanocortin 1 receptor	NM_002386.2
*SLC16A5*	0.8911	0.9846	0.0935	0.9538	0.0297	Solute carrier family 16; member 5	NM_004695.2
*LRRC56*	0.8893	0.9552	0.0658	0.9305	0.0664	Hypothetical protein LOC115399	NM_198075.1
*SYTL1*	0.8890	0.9215	0.0325	0.8721	0.0337	Synaptotagmin‐like 1	NM_032872.1
*EPHX2*	0.8886	0.9217	0.0331	0.9083	0.0357	Epoxide hydrolase 2; cytoplasmic	NM_001979.4
*SLC44A2*	0.8838	0.9691	0.0854	0.9538	0.0297	CTL2 protein	NM_020428.2
*TMEM58*	0.8830	0.9340	0.0510	0.9219	0.0393	Transmembrane protein 58	NM_198149.1
*AARSD1*	0.8817	0.9436	0.0619	0.9001	0.0364	Hypothetical protein LOC80755	NM_025267.2
*CCND1*	0.8793	0.9226	0.0432	0.8217	0.0349	Cyclin D1	NM_053056.1
*GRB7*	0.8777	0.9499	0.0722	0.9036	0.1166	Growth factor receptor‐bound protein 7	NM_005310.2
*CLSTN1*	0.8743	0.9028	0.0285	0.8493	0.0249	Calsyntenin 1 isoform 2	NM_014944.3

Methylation values for each CpG locus are expressed as a *β*‐value. Difference in KMX(−) − P(−): *β*‐value for K562/MX2 cells minus *β*‐value for K562/P cells without 5‐Aza‐2′‐deoxycytidine treatment.

KMX(−). Beta: *β*‐value for K562/MX2 cells without 5‐Aza‐2′‐deoxycytidine treatment.

P(−). Beta: *β*‐value for K562/P cells without 5‐Aza‐2′‐deoxycytidine treatment.

KMX(+). Beta: *β*‐value for K562/MX2 cells with 5‐Aza‐2′‐deoxycytidine treatment.

P(+). Beta: *β*‐value for K562/P cells with 5‐Aza‐2′‐deoxycytidine treatment.

**Table 3:** Methylation profile in K562/MX2 cells and K562/P cells. List of 30 highly methylated genes in K562/P cells compared to K562/MX2 cells

Gene symbol	Difference KMX(−)‐P(−)	KMX(−). Beta	P(−).Beta	KMX(+). Beta	P(+).Beta	Description	GenBank accession
*FLJ14166*	−0.9012	0.0366	0.9378	0.0288	0.9418	Hypothetical protein LOC79616	NM_024565.4
*PAPPA*	−0.8892	0.0386	0.9279	0.0414	0.9397	Pregnancy‐associated plasma protein A preproprotein	NM_002581.3
*PLOD2*	−0.8723	0.0240	0.8963	0.0224	0.9177	Procollagen‐lysine; 2‐oxoglutarate 5‐dioxygenase 2 isoform a precursor	NM_182943.2
*KIT*	−0.8634	0.0489	0.9123	0.0382	0.8964	V‐kit Hardy–Zuckerman 4 feline sarcoma viral oncogene homolog precursor	NM_000222.1
*EPHB3*	−0.8384	0.1107	0.9491	0.1378	0.9605	Ephrin receptor EphB3 precursor	NM_004443.3
*TOLLIP*	−0.8312	0.0787	0.9099	0.0645	0.9028	Toll interacting protein	NM_019009.2
*CMKLR1*	−0.8311	0.0457	0.8769	0.0488	0.8738	Chemokine‐like receptor 1	NM_004072.1
*PLK2*	−0.8198	0.0780	0.8978	0.1627	0.9172	Polo‐like kinase 2	NM_006622.1
*TUBB6*	−0.8149	0.1164	0.9313	0.1080	0.9435	Tubulin; beta 6	NM_032525.1
*IMPACT*	−0.8127	0.0839	0.8966	0.0642	0.8878	Impact homolog	NM_018439.1
*HMGB3*	−0.8088	0.0302	0.8391	0.0455	0.8161	High‐mobility group box 3	NM_005342.2
*GAS1*	−0.7955	0.0544	0.8500	0.0566	0.8367	Growth arrest‐specific 1	NM_002048.1
*NRP2*	−0.7911	0.0467	0.8378	0.0877	0.8919	Neuropilin 2 isoform 2 precursor	NM_003872.2
*ONECUT1*	−0.7902	0.1143	0.9045	0.0799	0.9279	One cut domain; family member 1	NM_004498.1
*FLJ32130*	−0.7772	0.0316	0.8088	0.0699	0.8098	Hypothetical protein LOC146540	NM_152458.4
*PAM*	−0.7719	0.0897	0.8616	0.0783	0.8202	Peptidylglycine alpha‐amidating monooxygenase isoform b; preproprotein	
*PSD4*	−0.7697	0.1044	0.8742	0.0925	0.8898	Pleckstrin and Sec7 domain containing 4	NM_012455.2
*DNAJC6*	−0.7696	0.1047	0.8742	0.0824	0.8941	DnaJ (Hsp40) homolog; subfamily C; member 6	NM_014787.2
*C6orf145*	−0.7690	0.0152	0.7842	0.0191	0.8368	Hypothetical protein LOC221749	NM_183373.2
*SLC16A10*	−0.7599	0.0236	0.7835	0.0199	0.8055	Solute carrier family 16; member 10	NM_018593.3
*ADCY9*	−0.7540	0.0482	0.8023	0.0681	0.8739	Adenylate cyclase 9	NM_001116.2
*C1orf111*	−0.7478	0.1083	0.8561	0.0516	0.8136	Hypothetical protein LOC284680	NM_182581.1
*BCAS4*	−0.7350	0.0243	0.7593	0.0374	0.6988	Breast carcinoma amplified sequence 4 isoform c	NM_001010974.1
*DNAI1*	−0.7336	0.0525	0.7862	0.0470	0.8133	Dynein; axonemal; intermediate polypeptide 1	NM_012144.2
*SOX3*	−0.7334	0.1791	0.9125	0.1547	0.8720	SRY (sex determining region Y)‐box 3	NM_005634.2
*COBLL1*	−0.7317	0.0314	0.7631	0.0209	0.8136	COBL‐like 1	NM_014900.3
*LOC253012*	−0.7124	0.0638	0.7763	0.0467	0.8106	Hypothetical protein LOC253012 isoform 2	NM_198151.1
*PROKR1*	−0.7018	0.0871	0.7889	0.0719	0.8314	G protein‐coupled receptor 73	NM_138964.2
*JAKMIP1*	−0.7013	0.0486	0.7499	0.0449	0.8045	Multiple coiled‐coil GABABR1‐binding protein	NM_144720.2
*TSPAN18*	−0.6900	0.1457	0.8357	0.1431	0.8207	Tetraspanin 18 isoform 2	NM_130783.2

Methylation values for each CpG locus are expressed as a *β*‐value. Difference in KMX(−) − P(−): *β*‐value for K562/MX2 cells minus *β*‐value for K562/P cells without 5‐Aza‐2′‐deoxycytidine treatment.

KMX(−). AVG Beta: *β*‐value for K562/MX2 cells without 5‐Aza‐2′‐deoxycytidine treatment.

P(−). AVG Beta: *β*‐value for K562/P cells without 5‐Aza‐2′‐deoxycytidine treatment.

KMX(+). AVG Beta: *β*‐value for K562/MX2 cells with 5‐Aza‐2′‐deoxycytidine treatment.

P(+). AVG Beta: *β*‐value for K562/P cells with 5‐Aza‐2′‐deoxycytidine treatment.

### Gene expression patterns in drug‐sensitive and ‐resistant leukemic cells

After filtering, 22,409 genes were eligible for statistical analysis. The analysis identified 10,515 genes as differentially expressed between MX2‐sensitive and ‐resistant leukemia cells. Of these, 4896 showed higher expression, and 5619 showed lower expression in the drug‐resistant leukemia cells compared with those genes in the parent cells (Tables 4, 5). Lists of all expression profiles including full gene names and accession numbers are shown in Table S2. 5‐Aza‐2′‐deoxycytidine treatment induced changes in the expression of a few genes including an increase in low‐expression genes in K562/MX2 cells, but expression of most genes was not changed (Table 4, 5).

**Table 4:** Expression profile in K562/MX2 cells and K562/P cells. List of highly expressed genes in K562/P cells compared to K562/MX2 cells

Gene Symbol	Fold change ([KMX−]/[P−])	KMX(‐) Signal (normalized)	P(−) Signal (normalized)	KMX(+) Signal (normalized)	P(+) Signal (normalized)	Description	Entrez Gene ID	Genbank accession
*CTAG1A*	1/804.040	18.9461	15233.4490	44.4236	98.8608	*Homo sapiens* cancer/testis antigen 1A (CTAG1A), mRNA	246100	NM_139250
*DLK1*	1/676.138	110.0428	74404.1773	119.4851	72983.7166	*Homo sapiens* delta‐like 1 homolog (Drosophila) (DLK1), mRNA	8788	NM_003836
*VCX3A*	1/329.081	34.8629	11472.7370	158.0995	8514.7709	*Homo sapiens* variable charge, X‐linked 3A (VCX3A), mRNA	51481	NM_016379
*XAGE1A*	1/304.000	111.6045	33927.7938	159.4567	32875.3584	*Homo sapiens* X antigen family, member 1A (XAGE1A), transcript variant a, mRNA	653219	NM_001097592
*VCX*	1/280.399	27.8574	7811.1865	98.0080	6299.8074	*Homo sapiens* variable charge, X‐linked (VCX), mRNA	26609	NM_013452
*VCX2*	1/279.433	22.2797	6225.7115	101.9774	5343.0137	*Homo sapiens* variable charge, X‐linked 2 (VCX2), mRNA	51480	NM_016378
*FHL2*	1/202.499	35.0010	7087.6714	28.0545	6380.1560	*Homo sapiens* four and a half LIM domains 2 (FHL2), transcript variant 5, mRNA	2274	NM_001039492
*BEX4*	1/201.518	20.8857	4208.8693	20.1313	4470.3064	*Homo sapiens* brain expressed, X‐linked 4 (BEX4), mRNA	56271	NM_001127688
*HCLS1*	1/170.605	62.1245	10598.7536	166.5296	9431.3528	*Homo sapiens* hematopoietic cell‐specific Lyn substrate 1 (HCLS1), mRNA	3059	NM_005335
*UCA1*	1/162.611	52.8277	8590.4024	45.0836	6804.6158	*Homo sapiens* urothelial cancer associated 1 (nonprotein coding) (UCA1), noncoding RNA	652995	NR_015379
*IFITM2*	1/150.728	69.2778	10442.1126	41.0087	9673.5787	*Homo sapiens* interferon induced transmembrane protein 2 (1‐8D) (IFITM2), mRNA	10581	NM_006435
*CYB5A*	1/136.984	53.3125	7302.9771	27.7678	7909.5779	*Homo sapiens* cytochrome b5 type A (microsomal) (CYB5A), transcript variant 2, mRNA	1528	NM_001914
*GPR68*	1/135.231	37.0398	5008.9309	15.6715	5059.3734	*Homo sapiens* G protein‐coupled receptor 68 (GPR68), mRNA	8111	NM_003485
*CD24*	1/118.205	63.0017	7447.1309	52.7524	8239.4025	*Homo sapiens* CD24 signal transducer mRNA, complete cds and 3′ region.	1E+08	L33930
*KIAA1324L*	1/116.956	19.5615	2287.8536	26.6255	2145.0269	*Homo sapiens* KIAA1324‐like (KIAA1324L), transcript variant 2, mRNA	222223	NM_152748
*MAGEC1*	1/90.8075	49.9079	4532.0090	105.6107	4763.7946	*Homo sapiens* melanoma antigen family C, 1 (MAGEC1), mRNA	9947	NM_005462
*LAPTM5*	1/89.9161	57.8335	5200.1625	46.0495	6055.6957	*Homo sapiens* lysosomal protein transmembrane 5 (LAPTM5), mRNA	7805	NM_006762
*PAGE1*	1/88.9132	18.0740	1607.0165	20.9886	1928.6839	*Homo sapiens* P antigen family, member 1 (prostate associated) (PAGE1), mRNA	8712	NM_003785
*CD33*	1/85.8366	22.5623	1936.6734	15.1992	1370.6828	*Homo sapiens* CD33 molecule (CD33), transcript variant 1, mRNA	945	NM_001772
*APOE*	1/85.4381	328.2242	28042.8431	371.4469	13980.5646	*Homo sapiens* apolipoprotein E (APOE), mRNA	348	NM_000041
*PLCD1*	1/85.1325	75.8706	6459.0488	90.9223	6264.9575	*Homo sapiens* phospholipase C, delta 1 (PLCD1), transcript variant 2, mRNA	5333	NM_006225
*CD86*	1/83.0913	108.8506	9044.5437	28.5954	271.0742	*Homo sapiens* CD86 molecule (CD86), transcript variant 2, mRNA	942	NM_006889
*HMBOX1*	1/75.5381	63.1101	4767.2217	19.9192	117.0972	Homeobox containing 1	79618	
	1/74.9294	33.2111	2488.4885	31.8588	1932.3772	1EA8_A chain A, apolipoprotein E3 22 kd fragment Lys146glu mutant.		BU194531
*VIL1*	1/74.6514	25.1293	1875.9400	40.8610	2293.2978	*Homo sapiens* villin 1 (VIL1), mRNA	7429	NM_007127
*FERMT3*	1/72.2681	27.4911	1986.7312	71.7283	2664.9782	*Homo sapiens* fermitin family homolog 3 (Drosophila) (FERMT3), transcript variant URP2LF, mRNA	83706	NM_178443
	1/67.7961	60.9207	4130.1873			*Homo sapiens* cDNA clone IMAGE:277235 5′, mRNA sequence		N47124

Consider “italicizing *Homo sapiens*” in the tables.

Normalized values for each gene are shown.

Fold change ([KMX−]/[P−]): Normalized value in K562/MX2 cells divided by normalized value in K562/P cells without 5‐Aza‐2′‐deoxycytidine treatment.

KMX(−) Signal (normalized): Normalized value in K562/MX2 cells without 5‐Aza‐2′‐deoxycytidine treatment.

P(−) Signal (normalized): Normalized value in K562/P cells without 5‐Aza‐2′‐deoxycytidine treatment.

KMX(+) Signal (normalized): Normalized value in K562/MX2 cells with 5‐Aza‐2′‐deoxycytidine treatment.

P(+) Signal (normalized): Normalized value in K562/P cells with 5‐Aza‐2′‐deoxycytidine treatment.

**Table 5:** Expression profile in K562/MX2 cells and K562/P cells. List of highly expressed genes in K562/MX2 cells compared to K562/P cells

Gene symbol	Fold change ([KMX−]/[P−])	KMX(−) Signal (normalized)	P(−) Signal (normalized)	KMX(+) Signal (normalized)	P(+) Signal (normalized)	Description	Entrez GeneID	Genbank accession
*HBB*	258.9561	53109.6352	205.0914	61532.7564	234.5827	*Homo sapiens* hemoglobin, beta (HBB), mRNA	3043	NM_000518
*PLAT*	77.6454	3227.8347	41.5715	2953.7541	35.6801	*Homo sapiens* plasminogen activator, tissue (PLAT), transcript variant 1, mRNA	5327	NM_000930
	72.2131	4016.1516	55.6153	1469.0889	52.5098	BF213738 601847628F1 NIH_MGC_55 *Homo sapiens* cDNA clone IMAGE:4078519 5′, mRNA sequence		BF213738
*CLEC2B*	66.8582	3750.5358	56.0969	1372.3312	58.6276	*Homo sapiens* C‐type lectin domain family 2, member B (CLEC2B), mRNA	9976	NM_005127
*CXCL1*	64.9437	2178.4647	33.5439	630.2581	45.0387	*Homo sapiens* chemokine (C‐X‐C motif) ligand 1 (melanoma growth stimulating activity, alpha) (CXCL1), mRNA	2919	NM_001511
*S100A10*	46.6281	14773.7249	316.8413	7658.6691	352.0523	*Homo sapiens* S100 calcium binding protein A10 (S100A10), mRNA	6281	NM_002966
*EMILIN2*	46.4009	16469.0418	354.9296	9837.7386	371.1331	*Homo sapiens* elastin microfibril interfacer 2 (EMILIN2), mRNA	84034	NM_032048
*PPFIBP1*	45.6525	1550.9474	33.9729	645.4800	26.4176	*Homo sapiens* PTPRF interacting protein, binding protein 1 (liprin beta 1) (PPFIBP1), transcript variant 1, mRNA	8496	NM_003622
*COL6A1*	43.9149	1951.9319	44.4480	1941.1771	37.1725	*Homo sapiens* collagen, type VI, alpha 1 (COL6A1), mRNA	1291	NM_001848
*IL8*	43.4885	2921.1090	67.1697	815.9359	65.7085	*Homo sapiens* interleukin 8 (IL8), mRNA	3576	NM_000584
*BATF3*	41.7773	5358.6282	128.2666	2401.9560	140.0571	*Homo sapiens* basic leucine zipper transcription factor, ATF‐like 3 (BATF3), mRNA	55509	NM_018664
*PGLYRP4*	41.3142	1049.9370	25.4135	721.4657	19.6105	*Homo sapiens* peptidoglycan recognition protein 4 (PGLYRP4), mRNA	57115	NM_020393
*FN1*	40.5872	1469.2060	36.1987	2108.7846	57.9448	*Homo sapiens* fibronectin 1 (FN1), transcript variant 1, mRNA	2335	NM_212482
*OSBPL6*	33.8573	1769.1553	52.2533	1159.4035	65.3581	Oxysterol binding protein‐like 6	114880	AK123248
*ZDHHC11*	32.7875	1003.1447	30.5954	1332.7551	42.2168	*Homo sapiens* zinc finger, DHHC‐type containing 11 (ZDHHC11), mRNA	79844	NM_024786
*TSPAN5*	32.5284	1956.8982	60.1596	1961.2544	106.6336	*Homo sapiens* tetraspanin 5 (TSPAN5), mRNA	10098	NM_005723
*TPST1*	29.7643	1097.9944	36.8900	918.5039	49.7456	*Homo sapiens* tyrosylprotein sulfotransferase 1 (TPST1), mRNA	8460	NM_003596
*CTSL2*	28.0364	3720.2736	132.6945	2890.063	135.4341	*Homo sapiens* cathepsin L2 (CTSL2), mRNA	1515	NM_001333
*PHLDA1*	27.7600	5847.4504	210.6430	2267.6291	92.4228	*Homo sapiens* pleckstrin homology‐like domain, family A, member 1 (PHLDA1), mRNA	22822	NM_007350
*C20orf56*	27.7375	2084.2000	75.1403	1494.8821	85.9847	*Homo sapiens* chromosome 20 open reading frame 56 (C20orf56), noncoding RNA	140828	NR_001558
*PLOD2*	26.9148	1185.7967	44.0574	1011.9656	30.4028	*Homo sapiens* procollagen‐lysine, 2‐oxoglutarate 5‐dioxygenase 2 (PLOD2), transcript variant 1, mRNA	5352	NM_182943
*SLC44A1*	26.5518	3707.7802	139.6434	973.0105	61.0888	*Homo sapiens* solute carrier family 44, member 1 (SLC44A1), mRNA	23446	NM_080546
*BACH2*	25.9313	605.3930	23.3461	321.7125	25.6866	*Homo sapiens* BTB and CNC homology 1, basic leucine zipper transcription factor 2 (BACH2), transcript variant 1, mRNA	60468	NM_021813
*HBD*	24.9633	8707.2836	348.8031	33730.3461	316.6115	*Homo sapiens* hemoglobin, delta (HBD), mRNA	3045	NM_000519

Normalized values for each gene are shown.

Fold change ([KMX−]/[P−]): Normalized value in K562/MX2 cells divided by normalized value in K562/P cells without 5‐Aza‐2′‐deoxycytidine treatment.

KMX(−) Signal (normalized): Normalized value in K562/MX2 cells without 5‐Aza‐2′‐deoxycytidine treatment.

P(−) Signal (normalized): Normalized value in K562/P cells without 5‐Aza‐2′‐deoxycytidine treatment.

KMX(+) Signal (normalized): Normalized value in K562/MX2 cells with 5‐Aza‐2′‐deoxycytidine treatment.

P(+) Signal (normalized): Normalized value in K562/P cells with 5‐Aza‐2′‐deoxycytidine treatment.

### Integrated methyl array data and expression array data

Next, we integrated the data from the methyl array and expression array to identify genes with strong methylation‐specific changes in expression after quantile normalization. After integration, 9596 genes were eligible for further analysis. These genes were classified into four groups (hypermethylated with lower expression, hypermethylated with higher expression, hypomethylated with higher expression, hypomethylated with lower expression). We identified 326 genes with hypermethylation and lower expression in K562/MX2 cells (Table S3A), 173 genes with hypermethylation and higher expression in K562/MX2 cells (Table S3B), 71 genes with hypomethylation and higher expression in K562/MX2 cells (Table S3C), and 61 genes with hypomethylation and lower expression in K562/MX2 cells (Table S3D).

### GO analysis in drug‐sensitive and ‐resistant leukemic cells

GO analysis was performed using the dataset for 631 genes with either significantly higher or lower expression combined with higher or lower methylation in drug‐resistant leukemia cells (K562/MX2) compared with drug‐sensitive leukemia cells (K562/P) (Table S4A). In K562/MX2 cells that responded poorly to MX2, etoposide, and doxorubicin, selective enrichment of genes with significantly altered expression and methylation was found in ontology categories related to the response to stimuli, gene silencing, the extracellular region, and the immune response (Table 6, Table S4A, B). The methylation and expression status of each gene indicated that half of the genes were highly methylated with lower expression in resistant cells (Table S4B).

**Table 6:** Analysis of 30 significant GO terms

GO identifier	GO term	Ontology	#Hits in group	Group size	#Hits expected	*P* value
GO:0050896	Response to stimulus	Biological process	244	5306	186	1.77776E‐06
GO:0070918	Production of small RNA involved in gene silencing	Biological process	62	876	31	2.98838E‐05
GO:0031047	Gene silencing by RNA	Biological process	62	885	31	3.07121E‐05
GO:0005576	Extracellular region	Cellular component	88	1437	51	3.52275E‐05
GO:0031050	dsRNA fragmentation	Biological process	62	876	31	3.73548E‐05
GO:0048583	Regulation of response to stimulus	Biological process	62	871	31	4.05668E‐05
GO:0070920	Regulation of production of small RNA involved in	Biological process	62	871	31	6.08503E‐05
GO:0043331	Response to dsRNA	Biological process	63	878	31	7.05792E‐05
GO:0016458	Gene silencing	Biological process	64	962	34	0.000104926
GO:0016020	Membrane	Cellular component	222	5069	178	0.000231564
GO:0002376	Immune system process	Biological process	104	1892	67	0.000282245
GO:0005886	Plasma membrane	Cellular component	168	3486	123	0.000335645
GO:0070887	Cellular response to chemical stimulus	Biological process	67	1070	38	0.000343859
GO:0006955	Immune response	Biological process	73	1208	43	0.000359805
GO:0042221	Response to chemical stimulus	Biological process	162	3330	117	0.000378179
GO:0032501	Multicellular organismal process	Biological process	254	6016	211	0.000403796
GO:0006952	Defense response	Biological process	71	1182	42	0.000572204
GO:0005737	Cytoplasm	Cellular component	233	5640	198	0.00324465
GO:0048518	Positive regulation of biological process	Biological process	157	3363	118	0.00379661
GO:0050776	Regulation of immune response	Biological process	30	384	14	0.00390334
GO:0009986	Cell surface	Cellular component	23	260	10	0.00402714
GO:0046649	Lymphocyte activation	Biological process	37	525	19	0.00417652
GO:0042110	T‐cell activation	Biological process	29	372	14	0.00430733
GO:0009611	Response to wounding	Biological process	62	1078	38	0.0060105
GO:0046651	Lymphocyte proliferation	Biological process	19	202	8	0.0060727
GO:0010033	Response to organic substance	Biological process	94	1836	65	0.00627684
GO:0044421	Extracellular region part	Cellular component	55	925	33	0.00629094
GO:0070661	Leukocyte proliferation	Biological process	19	204	8	0.00639204
GO:0002697	Regulation of immune effector process	Biological process	18	185	7	0.00653073
GO:0032943	Mononuclear cell proliferation	Biological process	19	204	8	0.00664772

GO, Gene Ontology.

### Key node search

Using the key node search, we found that p38*α* was an important factor in methylation‐related MX2 resistance.

### Increased phosphorylated p38*α* protein in MX2‐resistant leukemia cells and decreased phosphorylated p38*α* protein after pretreatment with p38*α* MAPK inhibitors

**Figure 1:**
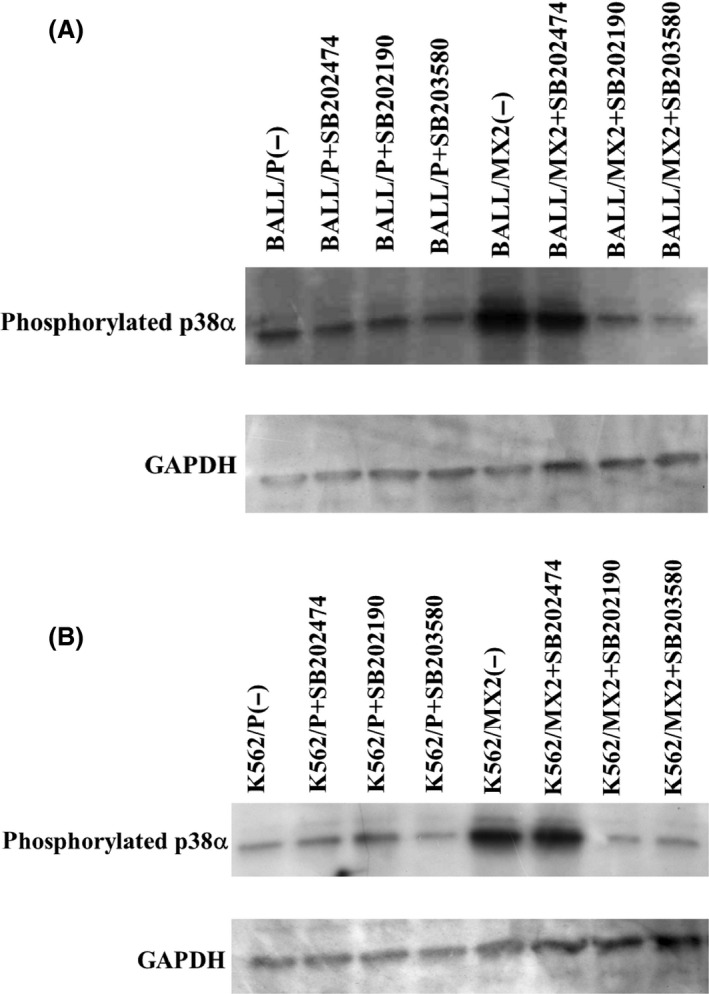
Increased phosphorylated p38*α* protein in MX2‐resistant leukemia cells and decreased phosphorylated p38*α* protein after pretreatment with p38*α* MAPK inhibitors. BALL/P, BALL/MX2, K562/P, and K562/MX2 cells were pretreated with or without SB202190, SB203580, or SB202474 for 30 min, and then phosphorylated p38 protein expression was measured. Phosphorylated p38*α* protein was increased in MX2‐resistant cells compared to parent cells. Specific inhibitors of p38 MAPK, SB203580 and SB202190, effectively decreased phosphorylated p38*α* protein in MX2‐resistant leukemia cells (K562/MX2, BALL/MX2). Representative data from three independent experiments are shown. MAPK, mitogen‐activated protein kinase.

We examined the expression levels of phosphorylated p38*α* protein in MX2‐resistant leukemia cells compared with sensitive parent cells (Fig. 1). Phosphorylated p38*α* protein was increased in MX2‐resistant cells compared to parent cells. The specific inhibitors of p38 MAPK, SB203580 and SB202190, effectively decreased the levels of phosphorylated p38*α* protein in MX2‐resistant leukemia cells (K562/MX2, BALL/MX2) (Fig. 1).

### Increased p38 kinase activity in MX2‐resistant leukemia cells and decreased p38 kinase activity after pretreatment with p38 MAPK inhibitors

**Figure 2:**
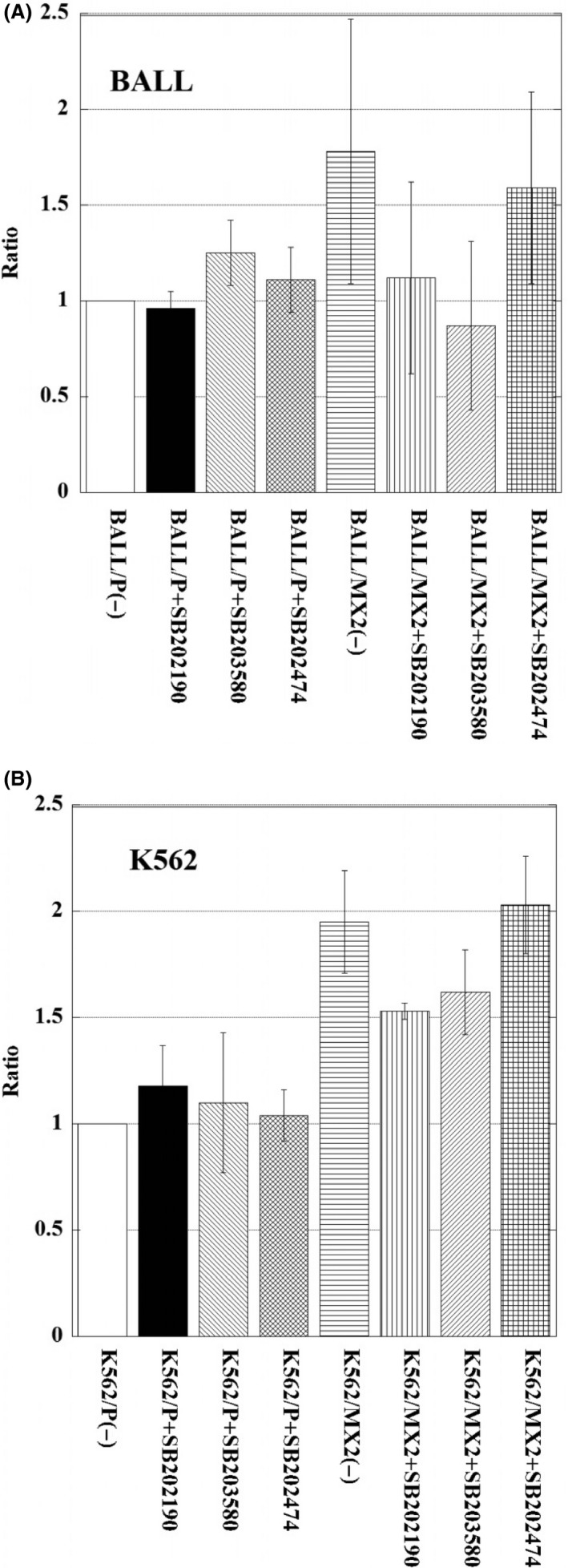
p38 kinase activity in BALL/P, BALL/MX2, K562/P, and K562/MX2 cells with or without pretreatment with SB202190, SB203580, or SB202474. BALL/P, BALL/MX2, K562/P, and K562/MX2 cells were pretreated with or without SB202190, SB203580, or SB202474 for 30 min, and then p38 kinase activity was measured. A significant increase in p38 kinase activity was observed in BALL/MX2 and K562/MX2 cells compared with BALL/P and K562/P cells, respectively. Treatment with SB202190 or SB203580 significantly decreased p38 kinase activity in BALL/MX2 and K562/MX2 cells compared with untreated BALL/MX2 and K562/MX2 cells, respectively. Data are the means ± standard deviation from three independent experiments. *P* < 0.05: BALL/MX2(−) versus BALL/MX2+ SB202190. *P* < 0.01: BALL/MX2(−) versus BALL/MX2+ SB203580. *P* < 0.005: BALL/MX2+ SB202474 versus BALL/MX2 +  SB202190. *P* < 0.02: BALL/MX2+ SB202474 versus BALL/MX2+ SB203580. *P* < 0.005: K562/MX2(−) versus K562/MX2 +  SB202190. *P* < 0.04: K562/MX2(−) versus K562/MX2 +  SB203580. *P* < 0.005: K562/MX2 +  SB202474 versus K562/MX2 +  SB202190. *P* < 0.001: K562/MX2 +  SB202474 versus K562/MX2 +  SB203580.

We next examined the p38 kinase activity in MX2‐resistant leukemia cells compared with sensitive parent cells (Fig. 2A and B). The p38 kinase activity was increased in MX2‐resistant cells compared to parent cells. Using specific inhibitors of p38 MAPK, SB203580 and SB202190, effectively decreased the p38 kinase activity in MX2‐resistant leukemia cells (K562/MX2, BALL/MX2), but SB202474 (negative control) did not decrease the activity (Fig. 2A and B).

### Increased cytotoxicity with MX2 in MX2‐resistant leukemia cells following pretreatment with p38 MAPK inhibitors

**Figure 3:**
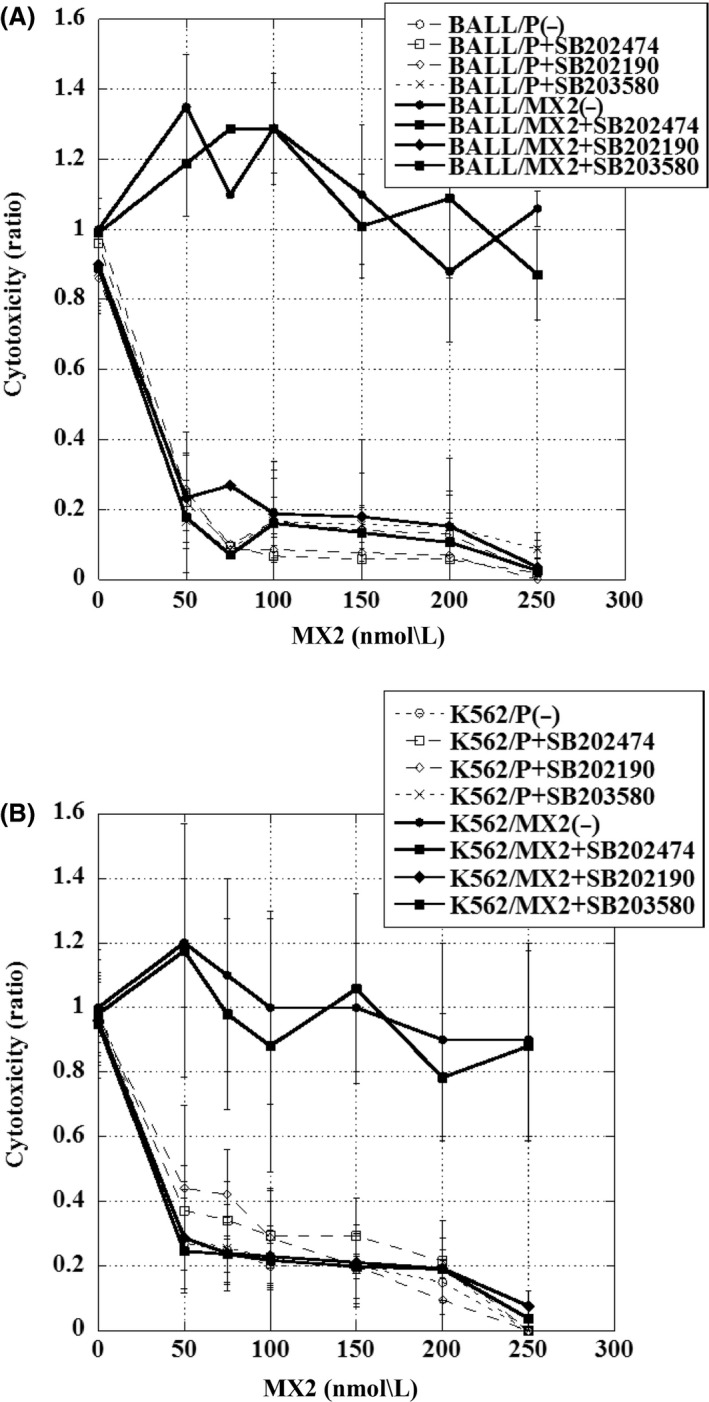
Cytotoxicity of MX2 in BALL/P, BALL/MX2, K562/P, and K562/MX2 cells with or without pretreatment with SB202190, SB203580, or SB202474. BALL/P, BALL/MX2, K562/P, and K562/MX2 cells were pretreated with or without SB202190, SB203580, or SB202474 for 30 min, and then treated with or without various concentrations of MX2 for 72 h. We showed graph at the 1 as without inhibitors and MX2 treatment. Slight decreased viability in BALL and K562 cells treated with SB202190, SB203580 without MX2 treatment. A significant increase in cytotoxicity with MX2 was observed in BALL/MX2 and K562/MX2 cells pretreated with SB202190 or SB203580. Data are the means ± standard deviation from three independent experiments.

First, we determined the optimal concentration of SB202190, SB203580, and SB202474 in K562 and BALL cells. Leukemia cells were incubated with various concentrations of these drugs for 72 hours, and the viability was measured. The IC50 values in BALL/P cells for SB202190, SB203580, and SB202474 were 400 ± 123 *μ*mol/L, 200 ± 6 *μ*mol/L, and 380 ± 88 *μ*mol/L, and in BALL/MX2 cells were 1200 ± 320 *μ*mol/L, 190 ± 67 *μ*mol/L, and 700 ± 267 *μ*mol/L, respectively. The IC50 values in K562/P cells were 560 ± 160 *μ*mol/L, 240 ± 62 *μ*mol/L, and 980 ± 80 *μ*mol/L, and in K562/MX2 cells were 250 ± 57 *μ*mol/L, 220 ± 35 *μ*mol/L, and 1200 ± 378*μ*mol/L, respectively. Based on these values and a previous report (Planchard et al. 2012), we used 10 *μ*mol/L SB202190, 10 *μ*mol/L SB203580, and 10 *μ*mol/L SB202474 as a pretreatment for 30 min. Then, various concentrations of MX2 were added, and the cells were cultured for 72 h. SB202190 or SB203580 pretreatment significantly increased the cytotoxicity of MX2 in BALL/MX2 cells and K562/MX2 cells, but not in parent cells (Fig. 3A and B). The combination index values of MX2 plus SB202190, SB203580, or SB202474 were 0.99, 1.08, and 1.08 in BALL/P cells, 0.06, 0.10, and 1.01 in BALL/MX2 cells, 1.18, 0.99, and 0.92 in K562/P cells, and 0.18, 0.18, and 1.51 in K562/MX2 cells, respectively. These results strongly suggested that MX2 synergistically acted with SB202190 or SB203580 in MX2‐resistant cells, but MX2 did not act synergistically with SB202474 (negative control).

### Increased p38*α* mRNA expression in MX2‐resistant leukemia cells and decreased p38*α* mRNA expression after pretreatment with siRNAs to knock down p38*α* MAPK expression

**Figure 4:**
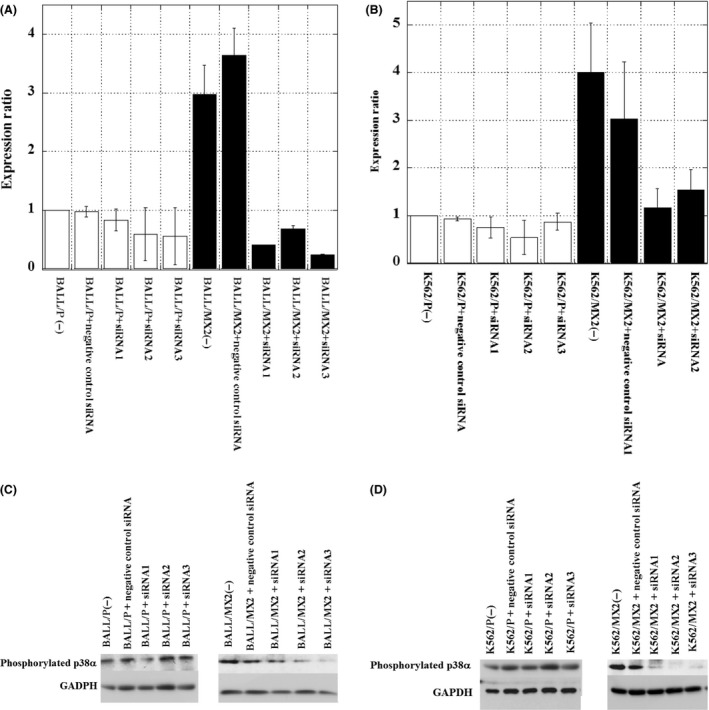
Increased p38*α* mRNA expression in MX2‐resistant leukemia cells and decreased p38*α* mRNA and protein expression after pretreatment with siRNAs to knock down p38*α* MAPK. (A and B) p38*α* mRNA expression was measured in MX2‐resistant leukemia cells and sensitive parent cells. p38*α* mRNA expression was increased in MX2‐resistant cells compared to parent cells. siRNAs (siRNA1, 2, 3) to knock down p38*α* effectively decreased p38*α* mRNA expression in MX2‐resistant leukemia cells (BALL/MX2(A), K562/MX2(B)). The data shown are from six independent experiments. *P* < 0.05: BALL/P versus BALL/MX2. *P* < 0.02: BALL/MX2(−) versus BALL/MX2 +  siRNA. *P* < 0.03: BALL/MX2(−) versus BALL/MX2 +  siRNA2. *P* < 0.01: BALL/MX2(−) versus BALL/MX2 +  siRNA3. *P* < 0.01: K562/P versus K562/MX2. *P* < 0.01: K562/MX2(−) versus K562/MX2 +  siRNA. *P* < 0.03: K562/MX2(−) versus K562/MX2 +  siRNA2. *P* < 0.01: K562/MX2(−) versus K562/MX2 +  siRNA3. (C) and (D) p38*α* protein was investigated in MX2‐resistant leukemia cells and sensitive parent cells. siRNAs (siRNA1, 2, 3) to knock down p38*α* effectively decreased p38*α* protein expression in MX2‐resistant leukemia cells (BALL/MX2(C), K562/MX2(D)). The data shown are representative data from two independent experiments. MAPK, mitogen‐activated protein kinase.

We next examined the p38*α* mRNA expression in MX2‐resistant leukemia cells compared with sensitive parent cells (Fig. 4). The p38*α* mRNA expression was increased in MX2‐resistant cells compared to parent cells. Using siRNAs (siRNA1, 2, 3) to knock down p38*α* effectively decreased the p38*α* mRNA and protein expression in MX2‐resistant leukemia cells (K562/MX2, BALL/MX2) (Fig. 4A–D).

### Increased cytotoxicity with MX2 in MX2‐resistant leukemia cells following pretreatment with siRNAs for p38*α* MAPK

**Figure 5:**
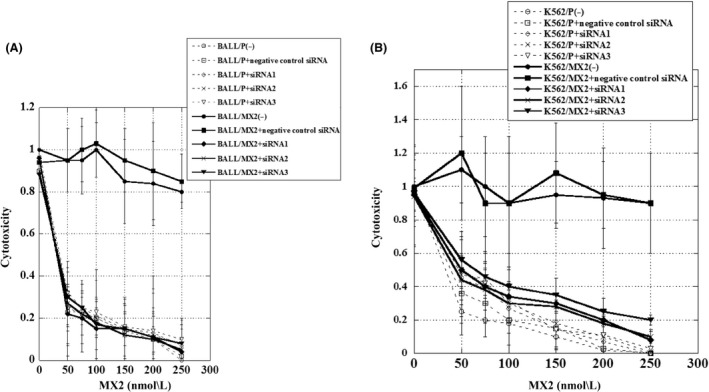
Cytotoxicity of MX2 in BALL/P, BALL/MX2, K562/P, and K562/MX2 cells with or without pretreatment with siRNA. BALL/P, BALL/MX2, K562/P, and K562/MX2 cells were pretreated with or without siRNA for 30 min, and then treated with or without various concentrations of MX2 for 72 h. Slight decreased viability in BALL and K562 cells treated with siRNA1, 2, 3 without MX2 treatment. A significant increase in cytotoxicity with MX2 was observed in BALL/MX2 and K562/MX2 cells pretreated with siRNA1, 2, 3. Data are reported as the mean ± standard deviation from three independent experiments. (A): BALL. (B): K562.

Pretreatment with siRNAs to knock down p38*α* mRNA expression significantly increased the cytotoxicity of MX2 in BALL/MX2 cells and K562/MX2 cells, but not in parent cells (Fig. 5). The combination index values of MX2 plus siRNA for knock down of p38*α* and negative control siRNA were 0.98, 0.96, and 0.99 for siRNA1, 2, 3, and 0.97 for negative control siRNA in BALL/P cells, 0.05, 0.04, and 0.08 for siRNA1, 2, 3, and 1.02 for negative control siRNA in BALL/MX2 cells, 0.98, 0.95, and 0.93 for siRNA1, 2, 3, and 0.95 for negative control siRNA in K562/P cells, and 0.15, 0.13, and 0.18 for siRNA1, 2, 3, and 0.97 for negative control siRNA in K562/MX2 cells, respectively. These results strongly suggested that MX2 synergistically acted with siRNAs in MX2‐resistant cells.

### CpG islands in p38*α* might contribute to changes in expression in MX2‐resistant leukemia cell lines

**Figure 6:**
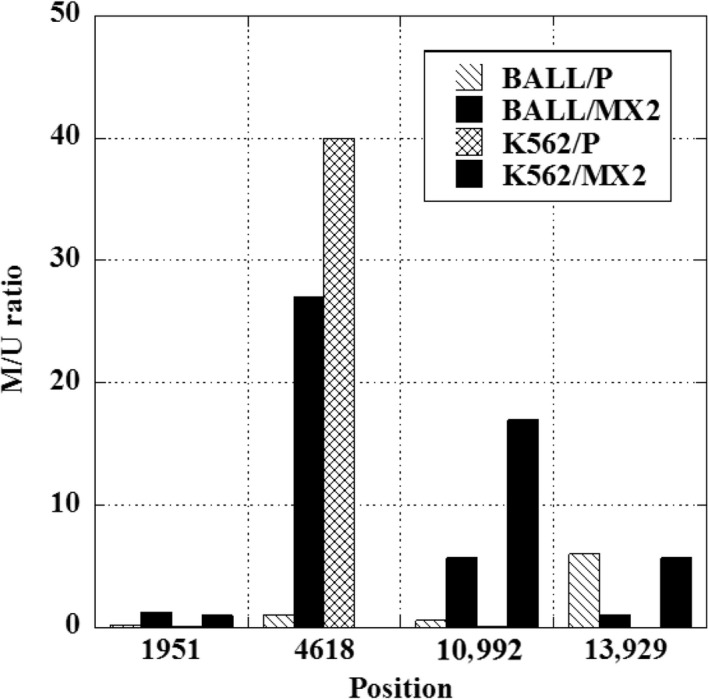
Methylation status in p38*α* in BALL, K562 parent cells, and MX2‐resistant cells. Methylated and unmethylated p38*α* gene was evaluated by methylation‐specific PCR using qPCR. Relative ratio was calculated as the expression with methylated primers/expression with unmethylated primers in each cell lines. Data are averages from two independent experiments.

We measured the methylation status in the p38*α* gene by MSP analysis in BALL/P, BALL/MX2, K562/P, and K562/MX2 cells. MX2‐resistant cell lines showed more methylation in p38*α* gene (position 1951, 10992 in BALL and K562, 4618 in BALL, 13929 in K562) we examined, but some of CpG islands (position 4618 in K562, and 13929 in BALL) showed more methylation in parent cells (Fig. 6).

### Caspase activity with SB202190, SB203580, and MX2 increased in MX2‐resistance leukemia cells

**Figure 7:**
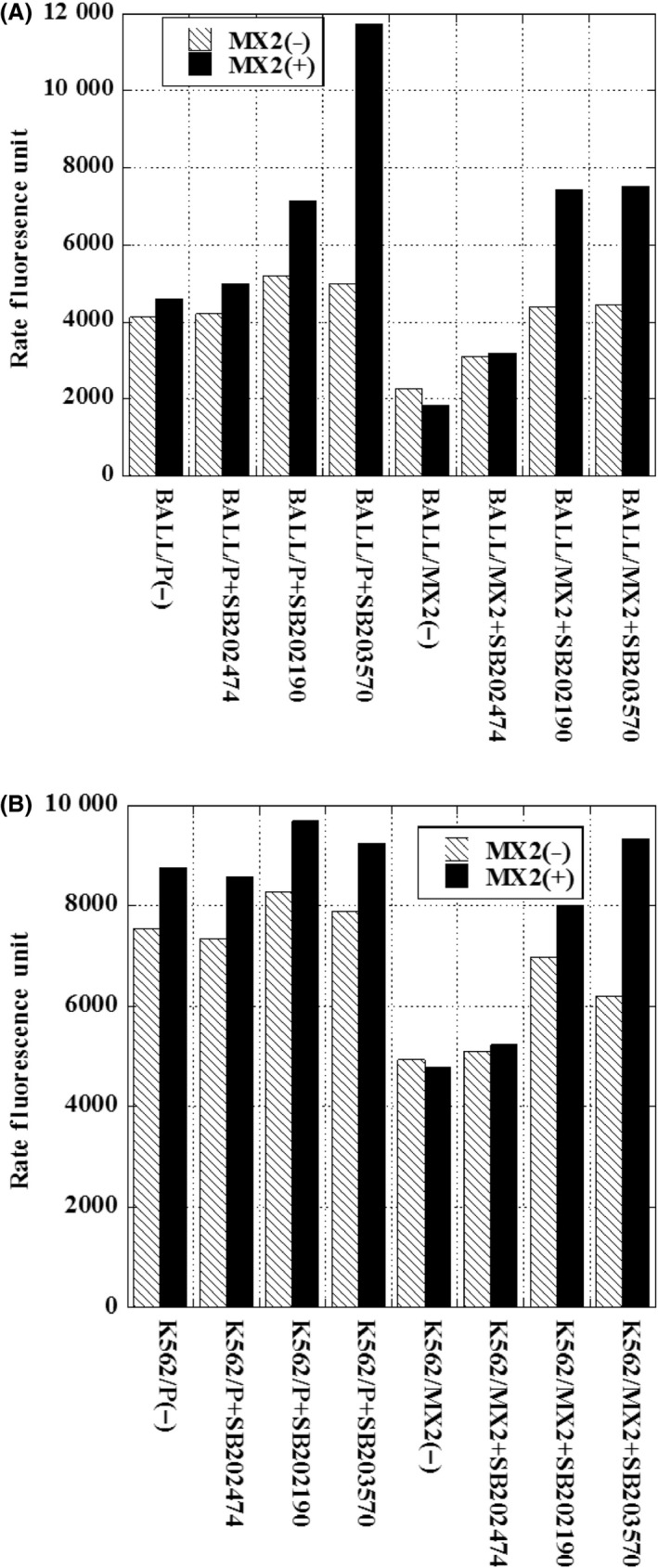
Caspase activities in BALL, K562 parent cells, and MX2‐resistant cells treated SB201290, SB203570, SB202474, and MX2. A total quantity of 10 *μ*mol/L SB202190, 10 *μ*mol/L SB203580, and 10 *μ*mol/L SB202474 were pretreated for 30 min. Then, 2.5 nmol/L of MX2 were added and incubated for 1 h and then caspase 3/7 activity assays were performed. Data are averages from two independent experiments. (A): BALL. (B): K562.

Caspase 3 (and caspase 7) showed decreased proteolytic activity in MX2‐resistant cells and slightly increased proteolytic activity at 30‐min exposure with SB202190 and SB203580. And, marked enhanced proteolytic activity was shown at 1 h exposure with 2.5 nmol/L of MX2 in MX2‐resistant cell lines treated with SB202190 and SB203580. (Fig. 7A and B).

## Discussion

Childhood leukemia is the most common childhood cancer. The majority of children can be cured with current therapies, although around 20% of children relapse, and their outcome remains dismal. Although reinduction regimens with higher doses of antileukemic drugs with or without stem cell transplantation are used to treat relapsed leukemia, the remission rate has not improved. Therefore, further dose intensification is not a viable option for improving outcomes, and other novel therapeutic options are necessary. Understanding the mechanism of drug resistance is essential prior to exploring new strategies against relapse. We previously reported that aberrant methylation of genes for key enzymes involved in drug metabolism is a novel mechanism of drug resistance (Asano et al. 2005). Exploitation of the methodology for analyzing the methylation status throughout the entire genome and the development of statistical methods for large datasets have enabled exploration and new insight into epigenetics and drug resistance (Hogan et al. 2011). Here, we found novel mechanisms involving p38*α* in resistance to MX2 using high‐throughput methylation analysis of multiple CpG sites and GO and key node analyses.

Inhibition of p38 MAPK activation by pharmacological inhibitors increased the cytotoxicity of MX2 in MX2‐resistant leukemia cell lines, but not in MX2‐sensitive cell lines. Our results suggest that adding p38 MAPK inhibitors will decrease the resistance to MX2 in MX2‐resistant leukemia cell lines by partially decreasing p38 activity.

The p38 MAPK pathway, which was initially identified as playing a role in stress and the inflammatory response, has a tumor suppressor function as well. The p38 MAPK pathway suppresses tumorigenesis by controlling the cell cycle, cell differentiation, cell proliferation, oncogene‐induced and replicative senescence, contact inhibition, the DNA damage response, and induction of apoptosis. p38 MAP kinase also inhibits apoptosis in several types of cells, including multiple myeloma cells (Navas et al. 2006; Wen et al. 2008). p38 activation mediates tamoxifen resistance in estrogen receptor‐positive breast tumors (Gutierrez et al. 2005). p38 inhibition enhances the sensitivity of multiple myeloma cells to arsenic trioxide and bortezomib (Wen et al. 2010). However, the details of the mechanism of enhanced drug sensitivity remain unclear. Our current study is the first to show that cytotoxicity due to increased inhibition of p38 MAPK is related to aberrant methylation in drug‐resistant leukemia cells.

The gold standard of current treatment against relapsed or refractory leukemia is allogeneic stem cell transplantation. This treatment strategy is effective for patients in complete remission, but not in patients who have not achieved complete remission. Although increasing the dose of cytotoxic drugs increases the cure rate and the rate of complete remission in relapsed patients, the cure rate has recently reached a plateau. A novel strategy to achieve complete remission in relapsed patients is needed. We believe that epigenetics is a major mechanism of drug resistance (Asano et al. 2005; Yamanishi et al. 2015). We analyzed the genome‐wide methylation status in MX2‐resistant leukemia cells and found that p38*α* was a key enzyme in MX2‐related drug resistance. This strategy for finding the key enzyme from the viewpoint of epigenetic changes may be a powerful concept for exploring new drugs to combat drug resistance. A p38 inhibitor may be a novel candidate for leukemia treatment.

In conclusion, our study showed that the p38*α* signaling pathway is involved in MX2‐induced drug resistance. Inhibition of p38 MAPK restored the sensitivity to MX2 in MX2‐resistant leukemia cell lines. Thus, p38 inhibitors may provide new chemotherapeutic options for overcoming drug resistance in the treatment of cancer. Further studies on the mechanisms of p38 inhibitors in drug resistance and the development of effective p38‐specific antagonists with low toxicity are expected to improve the clinical effects of chemotherapy.

## Author Contribution

Participated in research design: Takeshi Asano. Conducted experiments: Takeshi Asano, Hidehiko Narazaki, and Atsushi Fujita. Contributed new reagents or analytic tools: Takeshi Asano. Performed data analysis: Takeshi Asano. Wrote or contributed to the writing of the manuscript: Takeshi Asano.

## Disclosures

None declared.

## Supporting information


**Table S1.** Methylation profile of K562/P and K562/MX2 cells.
**Table S2.** Gene expression profile of K562/P and K562/MX2 cells.
**Table S3.** (A) List of genes with hypermethylation and lower expression in K562/MX2 cells. (B) List of genes with hypermethylation and higher expression in K562/MX2 cells. (C) List of genes with hypermethylation and higher expression in K562/MX2 cells. (D) List of genes with hypomethylation and lower expression in K562/MX2 cells.
**Table S4.** List of genes for Gene Ontology analysis. (B) Gene Ontology results and gene list.
